# The Influence of Mask Modification on Microneedle Strength in Deep X-Ray Lithography

**DOI:** 10.3390/mi17010136

**Published:** 2026-01-22

**Authors:** Jie Wang, Yigui Li, Lin Du

**Affiliations:** 1 School of Integrated Circuits and Communications, Suzhou Vocational Institute of Industrial Technology, Suzhou 215104, China; 01144@siit.edu.cn; 2 College of Science, Shanghai Institute of Technology, Shanghai 201418, China; 3 School of Mechanical Engineering, University of Shanghai for Science and Technology, Shanghai 200093, China

**Keywords:** LIGA processing, deep X-ray lithography, aligned exposure method, hollow microneedle array, photomask

## Abstract

Hollow microneedle arrays of different shapes were prepared for blood collection and precise drug delivery. This microneedle array was investigated using shape modification and hole position optimization, and different approaches to increase the strength of the microneedles and hole alignment were analyzed. Firstly, solid-tip microneedles were prepared using deep X-ray lithography, and an approach to increase the strength of microneedles by modifying the shape of the photomask was examined. Secondly, photomasks with holes in different positions were designed, and the exposure was aligned at different hole positions. Finally, the maximum stress and minimum displacement were analyzed using ANSYS 10.0 simulation software, while the proof-of-strength properties were accomplished by inserting microneedles into a polyimide film. The experimental results show that the modification of the shape of the photomask can increase the strength of the microneedles and compensate for the shortcomings generated by the moving exposure. Placing the holes away from the center of the tip can increase the flow rate of the microneedles. A horizontal offset of 30 μm and a vertical offset of 50 μm from the center of the microneedle tip were determined to be the best positions for aligning the holes. This meets the requirements for microneedle strength and sharpness.

## 1. Introduction

Biomedical MEMSs (microelectromechanical systems) represent one of the current hot research areas. Many miniaturized medical devices have been widely manufactured using this technology over the decades [1,2,3]. Conventional needles, traditionally used for blood collection and insulin injections, can damage the skin and cause a variety of chronic diseases. To overcome these problems, microneedles have become an important tool for connecting the organic matter underneath the human skin to external systems [4,5,6]. The outermost layer of the skin structure is the epidermis, which is approximately 100–150 μm in height. To extract blood from the skin, the tip of the microneedle needs to reach the capillaries in the dermis, which is located below the epidermis [7]. In the dermis, capillary density decreases closer to the epidermis. Consequently, microneedles with a height of approximately 250–500 μm are used, and the tips of the microneedles are sharp enough to be widely used as painless needles. Microneedles are mostly made of silicon, plastics, metals, and polymers, and they have various preparation methods, such as traditional silicon-based fabrication methods (wet etching and reactive ion etching) and micromolding using X-ray lithography methods [8,9,10]. The early silicon micromachining processes used for manufacturing MEMS devices possess inherent limitations, most notably a constrained selection of suitable processing materials. In contrast, the LIGA technique enables the processing of a diverse range of non-silicon materials, such as metals, ceramics, and polymers. Utilizing deep X-ray lithography (DXRL), the LIGA process can fabricate high-aspect-ratio microstructures, with heights reaching up to 1 mm and lateral dimensions as small as a few micrometers. In this study, high-aspect-ratio structures were first fabricated using synchrotron radiation-based X-ray lithography with a PMMA (poly(methyl methacrylate)) resist [11]. Subsequently, microneedle arrays were created via the planar pattern-to-cross-section transfer (PCT) technique [12]. Finally, hollow microneedles were produced using a hole photomask with an outer diameter of 100 μm and an inner diameter of 20 μm, employing different alignment strategies.

Currently, transdermal drug delivery primarily relies on four principal methods: gels, patches, iontophoresis, and microneedle technology. Microneedle technology, in particular, has emerged as a recent advancement for the delivery of certain compounds and is anticipated to be applicable to a broader range of agents in the near future. Solid microneedles, for instance, are used in acupuncture or integrated into DNA array chips. They facilitate drug delivery via coated surfaces and enable the detection of various physiological indicators by piercing the epidermis [13,14,15]. However, the delivered dose from solid microneedles is difficult to control precisely, as it depends on the number of cells in contact with the coated needle surface. In contrast, hollow microneedles, which can be connected to a reservoir via a through-hole, allow for precise control of drug dosage in delivery systems and are also suitable for blood sampling. Furthermore, they show potential in the development of implantable devices such as blood pressure sensors, microscopic bodily fluid analyzers, and surgical microscopy tools [16,17,18].

For the manufacturing of high-aspect-ratio microstructures, deep X-ray lithography (DXRL) has long been considered the gold standard. The pioneering work of Moon and Lee systematically detailed the principles and remarkable capabilities of DXRL, demonstrating its potential in fabricating structures with exceptionally high aspect ratios [19]. Nevertheless, its heavy dependence on synchrotron radiation sources has greatly restricted its widespread adoption and operational flexibility. To overcome this constraint, subsequent research has investigated various alternative methods. For instance, Khumpuang et al. developed an enhanced moving-mask lithography technique capable of achieving comparable structures under certain conditions, although the process involved multiple complex steps [20]. In another approach, Li et al. fabricated hollow polymer microneedle arrays from PMMA using a mask-dragging technique combined with X-ray lithography alignment, thereby underscoring the utility of this method for microneedle array production [21].

Beyond the inherent challenges in manufacturing, there is a growing and urgent application-driven need for high-performance hollow microstructures. This is especially evident in minimally invasive biomedical fields, such as the transdermal delivery of macromolecular drugs like insulin, where pump-free hollow microneedle patches have arisen as a highly promising platform [22]. In such systems, the lumen geometry of the microneedle critically influences drug-flow behavior and delivery efficiency, while the overall mechanical strength determines whether the needle can reliably penetrate the stratum corneum and withstand operational stresses—factors that directly affect the device’s safety and reliability. Consequently, the development of a fabrication process that can produce microstructures with complex internal cavities while retaining superior mechanical properties represents not only an advancement in micro-/nanofabrication technology, but also a key enabler for the practical implementation of such advanced medical devices.

In summary, existing technologies still face challenges in achieving flexible manufacturing of complex three-dimensional metal microstructures through a relatively simple process. To overcome these limitations, this paper proposes a method that leverages the influence of photomask shape on the geometry of solid microneedle arrays. Diverging from our previous study [10], the present work focuses on strengthening hollow microneedles via template-based shape modification and examines the effect of three distinct pore positions on microneedle strength. The core approach involves first fabricating solid microneedle arrays directly using the PCT technique, followed by producing hollow microneedle arrays via a hole mask alignment method, which allows precise definition of the pore position through photomask placement. By applying different pore alignment strategies, the exposure process of the photomask is iteratively optimized. Subsequently, the effectiveness of the shape modification method in enhancing microneedle strength and sharpness is evaluated. Finally, simulation combined with puncture experiments is conducted to verify that the fabricated hollow microneedle arrays satisfy the required strength criteria.

## 2. Materials and Methods

### 2.1. Design of Tip Microneedles

**Figure 1:**
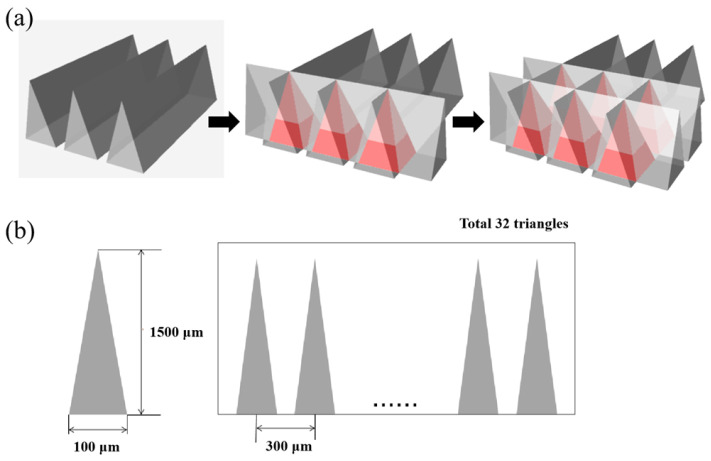
Photomask dimensions. (**a**) Fabrication of microneedles based on PCT technology. (**b**) Adjacent mask patterns with a center-to-center distance of 300 μm.

In this study, PMMA-tipped microneedles without internal flow channels were fabricated via deep X-ray lithography (DXRL) within the LIGA process, using an isosceles triangular photomask. Figure 1a illustrates the fabrication workflow of the microneedles based on planar pattern-to-cross-section transfer (PCT) technology. PCT is a method for shaping three-dimensional structures whose cross-sections correspond to the two-dimensional patterns on an X-ray mask. During exposure, the X-ray resist is translated repeatedly in upward and downward directions by a scanning stage while being irradiated with synchrotron radiation through the X-ray mask. The vertical movement of the stage gradually expands the exposed area in the PMMA, forming the microstructure. Since the X-ray absorption profile in the PMMA is determined by the absorber geometry on the mask, the cross-sectional shape defined on the mask is transferred into the resist, ultimately yielding a three-dimensional structure. The first and second X-ray exposures were performed at a 90° angle relative to each other. Figure 1b shows two adjacent mask patterns with a spacing of 300 μm. Each photomask contains 32 such patterns, enabling the fabrication of 1024 microneedles per unit area. The height of the microneedles is controlled by the exposure time and development duration, whereas the base dimensions remain unchanged when the same mask pattern is used.

**Figure 2:**
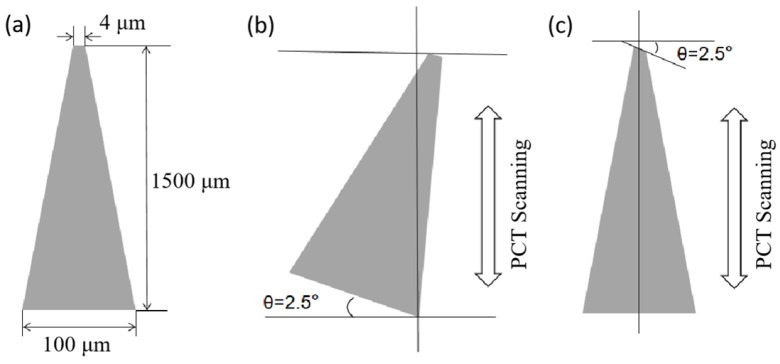
Three options for increasing strength. (**a**) Trapezoidal mask plate method. (**b**) Skewed exposure method. (**c**) Shape modification method.

Nevertheless, the triangular mask design yields microneedle arrays with critically low mechanical strength, due to the excessively narrow and steep geometry of the needle tips. As a first approach to address this issue, a trapezoidal mask can be employed instead of a triangular one, which produces microneedles with intentionally blunted tips (Figure 2a). Alternatively, a second solution involves tilting the X-ray mask during exposure (Figure 2b). The resulting microneedle tip fabricated with the trapezoidal mask measures approximately 100 nm in radius (or relevant dimension), while the optimized mask tilt angle was set to 2.5°. Although this technique produces stronger microneedles than those fabricated with a triangular mask pattern, their mechanical strength remains insufficient. To further enhance performance, a third strategy involves modifying the mask pattern from a trapezoidal to a polygonal design, as depicted in Figure 2c. This approach stems from the principle observed in the tilting-mask technique, where a sharp tip is formed despite the mask’s blunt geometry (e.g., trapezoidal), because the final three-dimensional structure results from the integration of the mask profile along the scanning direction. Thus, by switching to a polygonal mask pattern and adjusting the microneedle dimensions, both tip sharpness and overall strength are improved using the same fabrication platform. This design yields microneedles with sharp tips, increased mechanical robustness, and maintains shape integrity regardless of the tilt angle.

### 2.2. Design of Hollow Microneedles

**Figure 3:**
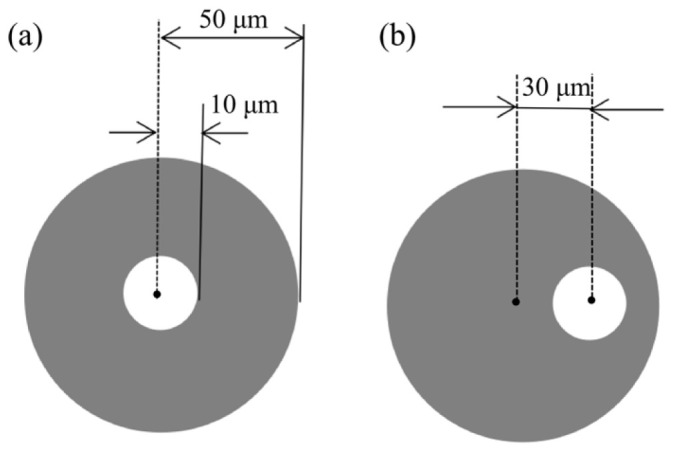
Hole photomask size and position. (**a**) At the center; (**b**) 30 μm off-center.

For dose-controlled drug delivery systems utilizing microneedles, an integrated fluid path—such as a microchannel or through-hole—is essential to enable controlled fluid transport. In microfabrication, through-holes in microneedles are typically created using tilted X-ray lithography. To facilitate this, a portable alignment fixture, which can be mounted temporarily onto the photoresist stage inside the X-ray exposure chamber, was employed to fabricate the microneedle through-holes. A through-hole with an inner diameter of 20 μm was incorporated at the tip of a 300 μm-long microneedle. The geometry and dimensions of the corresponding hole mask are illustrated in Figure 3.

Hollow microneedle arrays were fabricated using three distinct protocols that combined the planar pattern-to-cross-section transfer (PCT) technique with different hole-alignment strategies. The experimental design is summarized in Table 1. In Scheme 1, the through-hole was aligned to the center of the microneedle tip, and two PCT scans were carried out. Scheme 2 followed a similar procedure, except that the hole was offset by 30 µm from the tip center. Scheme 3 further streamlined the process by reducing the number of PCT scans from two to one. Since exposure cost scales with exposure time, the single-scan approach combined with through-hole patterning is expected to reduce both processing time and expense. In Scheme 3, the hole was likewise positioned 30 µm away from the tip center. All hole photomasks featured a pattern with an inner diameter of 20 μm and an outer diameter of 100 μm.

When fabricating hollow microneedles using the design approaches described above, Scheme 1 proves unsuitable for practical application. This is because the central through-hole can allow skin tissue to intrude during insertion, leading to clogging and rendering the design ineffective for fluid delivery. In contrast, Scheme 3, which employs a single through-hole exposure scan, is expected to reduce both processing time and cost, as exposure expense is directly proportional to exposure duration.

The design and optimization parameters for the three microneedle pore locations are summarized in Table 2. Placing the pore at the microneedle tip can lead to blunting and a reduction in effective needle length, which may cause tissue clogging during blood extraction. Conversely, positioning the pore away from the tip helps prevent clogging and improves fluid flow rate.

A pentagonal photomask, with a base length of 200 μm and a height of 500 μm, was designed to enhance the mechanical strength and tip sharpness of the microneedles. As noted earlier, three distinct hole-alignment strategies were implemented to optimize the pore position. Ultimately, by modifying the microneedle geometry and pore placement, this approach aims to fabricate hollow microneedles that are optimally suited for both drug delivery and blood collection applications.

## 3. Results

### 3.1. Preparation Process of Hollow Microneedles

The conventional DXRL method enables the fabrication of microstructures with high aspect ratios, offering advantages over other microfabrication techniques. However, it remains challenging to arbitrarily control wall structures into curved or sloped profiles. To improve controllability over wall shape, we therefore introduced the PCT method. By combining DXRL with PCT, microneedles can be formed with shapes closely matching the intended design, despite minor deviations caused by the development characteristics of PMMA and the GG developer (composed of 60 vol% 2-(2-butoxyethoxy)ethanol, 20 vol% tetra-hydro-1,4-oxazine, 5 vol% 2-aminoethanol-1, and 15 vol% purified water). This study focused on optimizing the hole photomask alignment by first fabricating solid microneedle tips using PCT, followed by creating hollow microneedles via aligned exposure. During alignment, an inexpensive aluminum fixture equipped with a manual X-Y-Z stage—primarily used for PMMA positioning—served as an adjustable PMMA holder (see Appendix A for details).

The total deposited dose (D, in ampere-hours) for microneedle structure exposure was determined using three parameters: the mask pattern height (H, in meters), the X-ray dose rate (d, in A·h/m), and the scan length of the resist stage (S, in meters). The relationship is given by Equation (1):

(1) $$D=d\cdot \left(\frac{S}{H}+1\right)$$

In this study, the microneedle mask pattern had a height of 1500 μm and a scan length of 9000 μm. To achieve a target process depth of approximately 300 μm, a dose of 0.06 A·h was required per unit depth. Consequently, the total deposited dose for each exposure was calculated to be 4.2 A·h. Because the PCT exposure for the microneedle was performed twice—at stage angles of 0° and 90°—the total dose for the double exposure amounted to 8.4 A·h, corresponding to an exposure time of approximately 5 h using synchrotron radiation (SR) from the AURORA (Ritsumeikan University, Japan) storage ring.

After exposure, the PMMA was developed in a GG developer for 3 h, followed by immersion in a stop solution for 10 min, and finally rinsed with purified water for 10 min. Throughout these steps, all chemicals were stirred at 300 rpm and maintained at 37 °C. The development temperature was set to 37 °C because the PMMA structure will subsequently serve as a master for nickel electroplating. The optimal temperature for a nickel-sulfamate bath is 37 °C, at a pH between 4 and 5 and under low current density. Temperatures significantly above or below this range induce higher internal stress in the PMMA master.

For the through-hole drilling process, an exposure dose of 0.1 A·h was applied. The exposed PMMA was then developed for 15 h, transferred to a stop solution for 10 min, and rinsed with deionized water for 5 min to complete the fabrication sequence.

**Figure 4:**
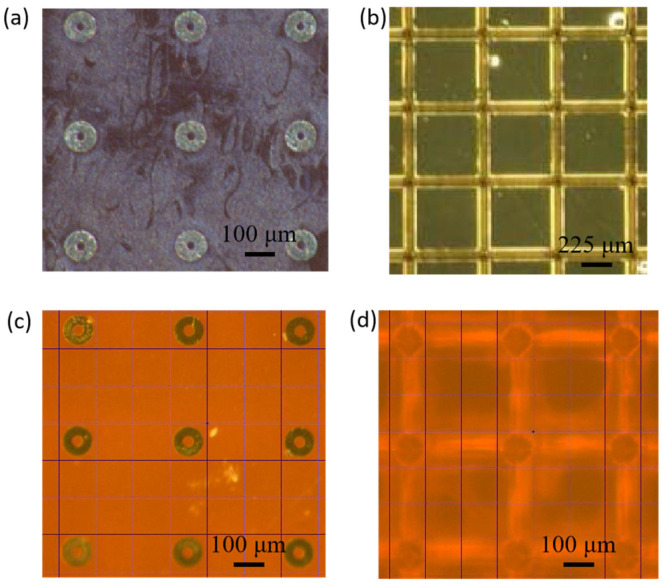
Top view of the schematic of the alignment procedure. (**a**) Positioned hole photomask. (**b**) Positioned tip microneedle array. (**c**) Alignment of microscope reticle frame with hole photomask. (**d**) Alignment of microscope reticle frame with tip microneedle.

A digital microscope (KEYENCE VH-8000, Osaka, Japan) was used to examine the fabricated structures. The reticle mode (frame function) was selected from the microscope software to assist in alignment. Before aligning the calibrated hole photomasks to the prefabricated tip microneedle arrays (made via PCT), the photomasks were first aligned to the microscope’s reticle frame by adjusting the stage. The relative alignment of the hole photomask, tip microneedle, and microscope reticle frame is illustrated in Figure 4. Due to slight stage movement after manual locking, the alignment stage exhibited a positional uncertainty of approximately ±3 μm.

### 3.2. Experimental Results of Tip Microneedles

**Figure 5:**
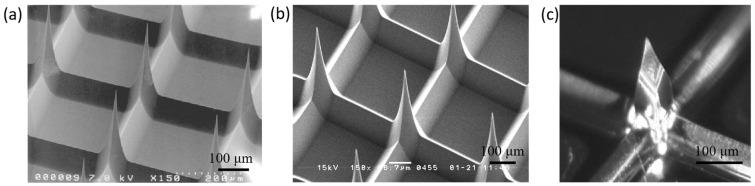
Microneedle arrays fabricated using (**a**) a triangular photomask; (**b**) a tilt-optimized trapezoidal photomask, and (**c**) a pentagonal photomask.

Figure 5a and Figure 5b show scanning electron microscopy (SEM) images of tapered microneedle arrays fabricated using a triangular photomask and a trapezoidal photomask (with an additional inclination angle of 2.5°), respectively. Figure 5c presents a magnified view of the microneedle tip fabricated with a pentagonal photomask, obtained using digital microscopy. The microneedles exhibit sharper tips as a result of the improved mask design.

### 3.3. Experimental Results of Hollow Microneedles

**Figure 6:**
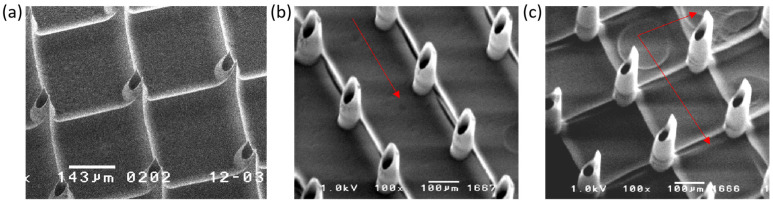
Hollow microneedle arrays fabricated with different channel positions and PCT scan strategies: (**a**) central channel (no offset); (**b**) channel offset by 30 μm from the tip center, produced with a single PCT scan; (**c**) channel offset by 30 μm from the tip center, produced with a double PCT scan. Red arrows indicate the scanning direction.

As summarized in Table 1, Protocol 1 produced microneedles with channels located at the tip center (Figure 6a), a configuration that was found to be less effective for blood sampling. Improved Protocols 2 and 3, employing single and double PCT scans, respectively, fabricated hollow microneedles whose channels were offset from the tip center, as illustrated in Figure 6b,c. Although both variants exhibited similar overall geometry, the double-scan protocol yielded sharper tips owing to enhanced structural integrity. The relatively incomplete polymerization associated with the single-scan approach, while more time- and cost-efficient, indicates that further optimization through geometric adjustment of the photomask pattern is still required.

**Figure 7:**
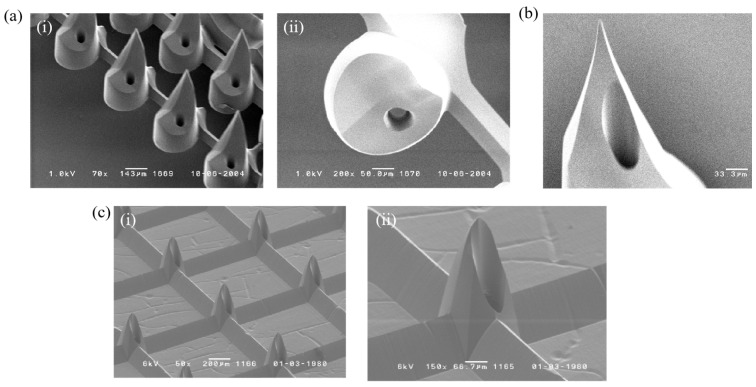
Microneedle arrays fabricated using different alignment protocols: (**a**) Alignment Protocol 1: (**i**) hollow microneedle array, (**ii**) top-view morphology of a single microneedle. (**b**) Alignment Protocol 2: tip of a single microneedle. (**c**) Alignment Protocol 3: (**i**) optimized hollow microneedle array, (**ii**) side-view of a single microneedle.

The hollow microneedles shown in Figure 7 measure approximately 300 μm in height, with a base diameter of 100 μm and a channel diameter of 20 μm. Further optimization of channel positioning will require systematic investigation. To improve mechanical robustness, the photomask geometry was modified from a triangular to a pentagonal design. This change yielded microneedles with an increased height of 350 μm. For refined channel placement, two photomask designs were implemented using three alignment protocols. As outlined in Table 2, alignment modes 1–3 correspond to the SEM images presented in Figure 7a–c, respectively.

### 3.4. Simulation and Strength Testing of Hollow Microneedles

All simulations were performed in ANSYS 10.0 for static structural analysis to optimize the channel mask design. The objective was to compare the stress and deformation responses of individual microneedles fabricated under different alignment protocols (Protocols 1, 2, and 3) under identical loading conditions. The model material was defined as a continuous, homogeneous, isotropic, linear elastic solid with a Young’s modulus of 3.2 GPa, a Poisson’s ratio of 0.37, and a yield strength of approximately 70 MPa. The bottom face of the microneedle was fully fixed, constraining all degrees of freedom. A concentrated axial force of 10 N was applied to the central node on the tip face to simulate a representative axial load encountered during skin penetration. A static linear analysis was carried out using an automatically generated mesh composed of higher-order tetrahedral elements.

**Figure 8:**
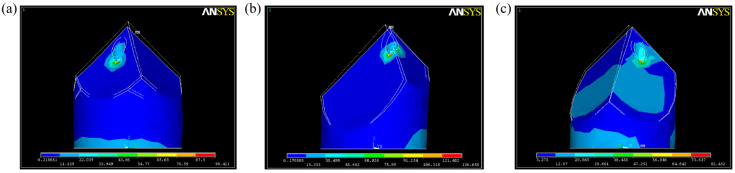
Simulation analysis results of stress and deformation. (**a**) Alignment 1. (**b**) Alignment 2. (**c**) Alignment 3.

The model was employed to assess the maximum stress and displacement of the microneedles during simulated penetration. Figure 8 presents the maximum stress sustained by the microneedle models under a constant load. Under a load of 10 N, the maximum stress in the optimized model from Alignment Protocol 3 reached 133.65 MPa. Since this value exceeds the material’s yield strength (~70 MPa), the linear elastic stress results are primarily used for qualitative comparison of relative mechanical performance among the designs. In comparison, the maximum stresses for Protocols 1 and 2 were 98.41 MPa and 82.43 MPa, respectively. The corresponding displacements after load application were 1.5 μm (Protocol 1), 6 μm (Protocol 2), and 3 μm (Protocol 3). These results indicate that microneedles fabricated using Alignment Protocol 3 withstand the highest stress while exhibiting the smallest displacement, confirming their superior mechanical robustness.

**Figure 9:**
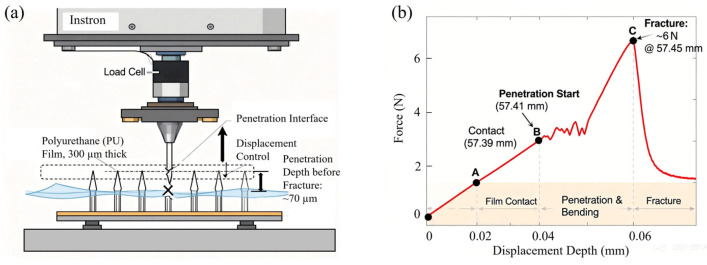
Mechanical strength characterization of hollow microneedle arrays. (**a**) Schematic of the experimental setup. (**b**) Force–displacement curve.

As shown in Figure 9a, strength testing was performed using a force-indentation instrument (Instron 2530, Osaka, Japan) on a polyurethane film simulating skin material. A set of 15 hollow microneedle arrays fabricated using Alignment Protocol 3 (see Table 2) was mounted on the indenter, which was then oriented upward to contact a polyurethane film with a thickness of 300 µm. The pressure sensor was activated at the film surface, and pressure was applied slowly to allow the needles to penetrate the film. The indenter moved downward at a constant quasi-static rate of 0.1 mm/min to ensure quasi-static loading and avoid dynamic effects. The load was measured by a 5 N load cell built into the machine with an accuracy of ±0.1% FS, and the displacement was recorded by the crosshead displacement sensor. The data acquisition frequency was set at 10 Hz. A sudden drop in the load was defined as the fracture of the microneedle structure.

Figure 9b shows the pressure load measurement results. The origin point is redefined as the instant the probe tip contacts the surface of the thin film. The graph indicates that the measurement started at 57.39 mm, where the load sensor contacted the surface of the polyurethane film. Microneedle penetration began at 57.41 mm. The needles fractured at 57.45 mm, corresponding to a total load of 6 N. The measured displacement was approximately 70 µm. Before fracture, the load borne by each individual needle was 0.4 N/needle.

**Figure 10:**
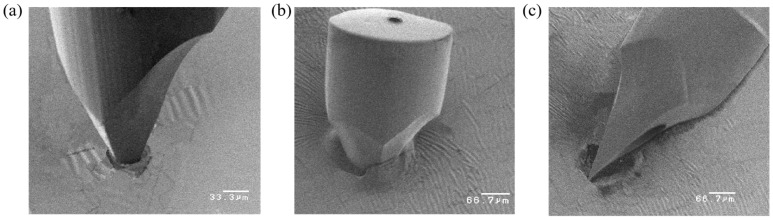
Penetration strength test of a microneedle into a polyimide film: (**a**) at 50 μm depth; (**b**) at 120 μm depth; (**c**) after load removal.

The mechanical strength of the microneedles was validated by inserting them into a polyimide film, with images captured during the penetration process, as shown in Figure 10. The micrographs demonstrate that the strength of the microneedles was effectively improved, as the tips remained fully intact after penetration.

## 4. Conclusions

In this study, tapered microneedles were fabricated using deep X-ray lithography (DXRL) within the LIGA process. By applying aperture-aligned exposure with different channel photomasks and alignment strategies, hollow microneedle arrays with distinct structural features were produced. The mechanical robustness of the needles was confirmed through successful insertion into polyimide films. To address the insufficient strength observed in microneedles produced with triangular photomasks, a pentagonal photomask was adopted, which also helped mitigate issues associated with the more cost-effective single-scan PCT process. Using two channel photomask designs and three alignment protocols, microneedles were eventually fabricated that resist clogging and promote higher flow rates. ANSYS simulations, supported by a low-cost alignment procedure, demonstrated that Alignment Protocol 3—featuring a horizontal offset of 30 μm and a vertical displacement of 50 μm from the tip center—achieved the highest stress resistance and mechanical strength. When connected to fluid reservoirs, the resulting hollow microneedles allow precise dosage control in blood extraction and drug delivery, and show promising potential for biomedical applications such as microcatheters and micromotors.

## Figures and Tables

**Table 1:** Experimental design for different PCT scan numbers and hole alignment strategies.

Scheme	No. of PCT Scans	Hole Photomask
1	2	Figure 3a
2	2	Figure 3b
3	1	Figure 3b

**Table 2:** Strategies for optimizing channel-aligned exposure in microneedle fabrication.

Optimization Protocol No.	Hole Photomask Pattern	Alignment	Schematic (Top View)
1	Figure 3b	Positioned at the center of the microneedle tip	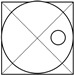
2	Figure 3a	Positioned 50 μm to the right of the microneedle tip center	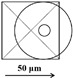
3	Figure 3b	Positioned 50 μm vertically below the microneedle tip center	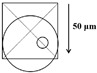

**Figure A1:**
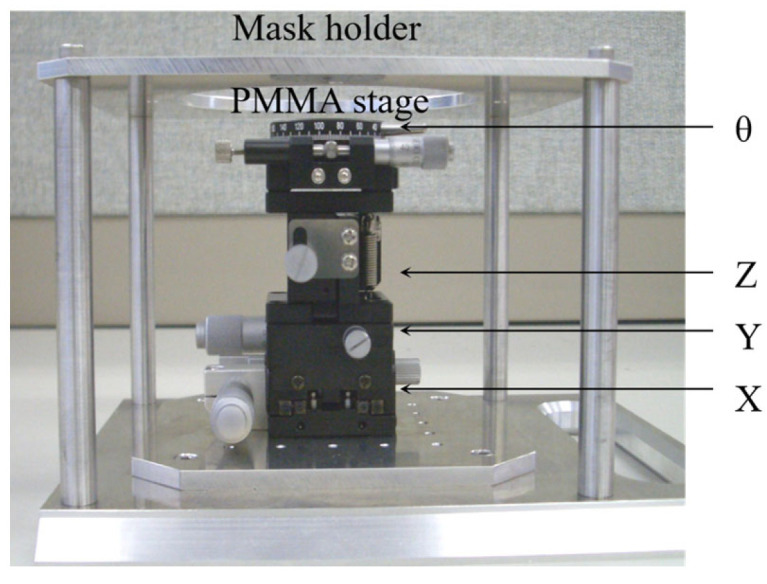
The low-cost alignment tool with a manual X-Y-Z stage.

## Data Availability

The original contributions presented in this study are included in the article. Further inquiries can be directed to the corresponding author.

## Abbreviations

The following abbreviations are used in this manuscript:

MEMS	Micro Electrical Mechanical System
PCT	Pattern-to-cross-section transfer
DXRL	Deep X-Ray Lithography
SR	Synchrotron Radiation
SEM	Scanning Electron Microscopy

## Appendix A

In the alignment process, a low-cost alignment tool made of aluminum was used as an adjustable PMMA stage. Figure A1 shows the alignment tool which consisted of a manual X-Y-Z stage for PMMA positioning. A digital microscope (KEYENCE, VH-8000, Osaka, Japan) was used with the frame function (mesh-scale mode) provided in the menu function of the microscope. The frame position remains unchanged while the microscope focus was adjusted. The hole mask was first aligned within each frame by adjusting the microscope lens position or by visual inspection; in practice, the frame was fitted to the mask pattern. The single-tip microneedle, previously fabricated using the PCT technique, was then aligned within the frame after focusing the lens on the microneedle array. The alignment stage had an alignment error of ±3 µm, which was small compared to the dimensions of the microneedles. This ±3 µm error was caused by slight stage shifting after manual locking.
